# Controllable synthesis and characterization of Mg_2_SiO_4_ nanostructures *via* a simple hydrothermal route using carboxylic acid as capping agent and their photocatalytic performance for photodegradation of azo dyes

**DOI:** 10.1039/d1ra02244j

**Published:** 2021-06-18

**Authors:** Saeed Moshtaghi, Masoud Hamadanian, Omid Amiri, Maryam Goli, Masoud Salavati-Niasari

**Affiliations:** Institute of Nano Science and Nano Technology, University of Kashan P. O. Box 87317-51167 Kashan I. R. Iran hamadani@kashanu.ac.ir salavati@kashanu.ac.ir +98 31 5555 29 30 +98 31 5591 2383; Faculty of Chemistry, Razi University P. O. Box 6714414971 Kermanshah Iran; Department of Chemistry, College of Science, University of Raparin Rania Kurdistan Region Iraq

## Abstract

Magnesium silicate (forsterite) nanoparticles were synthesized by a facile hydrothermal method, and characterized using several techniques such as XRD, SEM, EDS, DRS, Raman, TEM, and FT-IR. Several carboxylic acid structures were applied to modify the morphology and surface properties of the as-prepared particles. In this manuscript, citric acid, maleic acid, and succinic acid were used as the carboxylic acid agents. The effect of changing the ratio of carboxylic acid agent to central metal on the morphology and photocatalytic behavior was evaluated. The activities of the Mg_2_SiO_4_ nanostructures as photocatalysts were assessed by the degradation of several azo dyes (Acid Blue 92, Acid Brown 14, and Acid Violet 7) under UV and Vis light irradiation. The degradation percentages of Acid Blue 92 were about 88% and 74% in the presence of Vis and UV light respectively, and the percentages for photodegradation of Acid Brown 14 were approximately 76% and 82% in the presence of Vis and UV light, respectively. Furthermore, the degradation percentages for Acid Violet 7 were 93% and 80% under UV and Vis light, respectively.

## Abstract 2

Mg_2_SiO_4_ nanostructures have been synthesized *via* a facile hydrothermal approach. The photocatalytic behavior of Mg_2_SiO_4_ nanostructures prepared by different carboxylic acids have been investigated to degrade azo dyes under UV and visible light.

## Graphical abstract

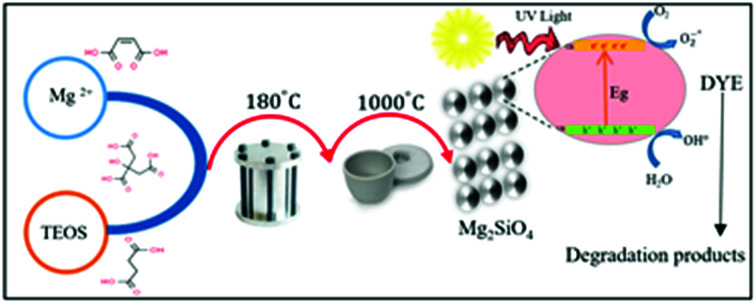

## 1. Introduction

Nowadays water pollution is one of the biggest problems for humankind. Different approaches have been used for water treatment, such as absorption,^1^ flocculation,^2^ membrane processes,^3^ and photocatalytic degradation.^4^ One of the significant industrial contaminants is organic and industrial dyes, which can be removed simply by photocatalytic degradation.^5–14^ Nanomaterials used widely in photocatalytic processes are the right candidates for this process, due to the chemical, physical, electrical, magnetic, optical, and mechanical properties.^15,16^

Among the nanomaterials, the silicates family is a noteworthy and unique inorganic nanomaterial. Silicates exhibit a variety of stoichiometric and crystal structures that are naturally plentiful.^17,18^ Forsterite (Mg_2_SiO_4_) is an essential sub-group of the silicate family with particular applications in several technologies such as long-lasting phosphor, X-ray imaging, light-emitting displays and environmental monitoring.^19–22^ Mg_2_SiO_4_ has been synthesized, preferably *via* solution-based methods to achieve excellent chemical homogeneity and small crystallite size compared to traditional solid-state reaction, which needs higher calcination temperature to gain pure phase crystals.^23^

A variety of synthesis approaches such as solid-state,^24^ sol–gel,^25^ combustion,^26^ and hydrothermal^27^ have been used for preparing forsterite. Amongst all methods as mentioned earlier, hydrothermal has obtained growing consideration owing to the full range of advantages such as the wide range of precursors, high purity product, easy experimental setup, low temperature, and shorter reaction time.^28^

In this paper, Mg_2_SiO_4_ nanoparticles have been prepared by the hydrothermal method and different carboxylic acids such as succinic acid, maleic acid, and citric acid were used as a capping agent. Afterward, the obtained products were calcined at defined temperatures, and characterized using several analysis techniques such as SEM, TEM, XRD, EDS, DRS, Raman and FT-IR. Finally the photocatalytic degradation of different azo dyes Acid Blue 92, Acid Violet 7, and Acid Brown 14 under UV and Vis irradiation was studied, as well.

## 2. Experimental

### 2.1 Materials and physical measurements

Generally, the chemicals for the obtaining of Mg_2_SiO_4_ nanostructures, including Mg(NO_3_)_2_·6H_2_O (98%), tetraethyl orthosilicate (TEOS) (99%), citric acid anhydrous, maleic acid and succinic acid were purchased from Merck and Sigma-Aldrich Company and used without further purification. Also, de-ionized water was used as the solvent. XRD patterns were recorded using a Philips X-ray diffractometer using Ni-filtered Cu Kα radiation. SEM images were taken using an LEO instrument model 1455VP. Before capturing images, the samples were coated by a thin layer of Au to make the sample surface a conductor and prevent charge accumulation to obtain a better contrast. FT-IR spectra were recorded on a Galaxy series FTIR 5000 spectrophotometer. The electronic spectra of the complexes were taken on a UV-visible scanning spectrometer (Shimadzu, UV-2550, Japan).

### 2.2 Preparation of Mg_2_SiO_4_

**Scheme 1:**
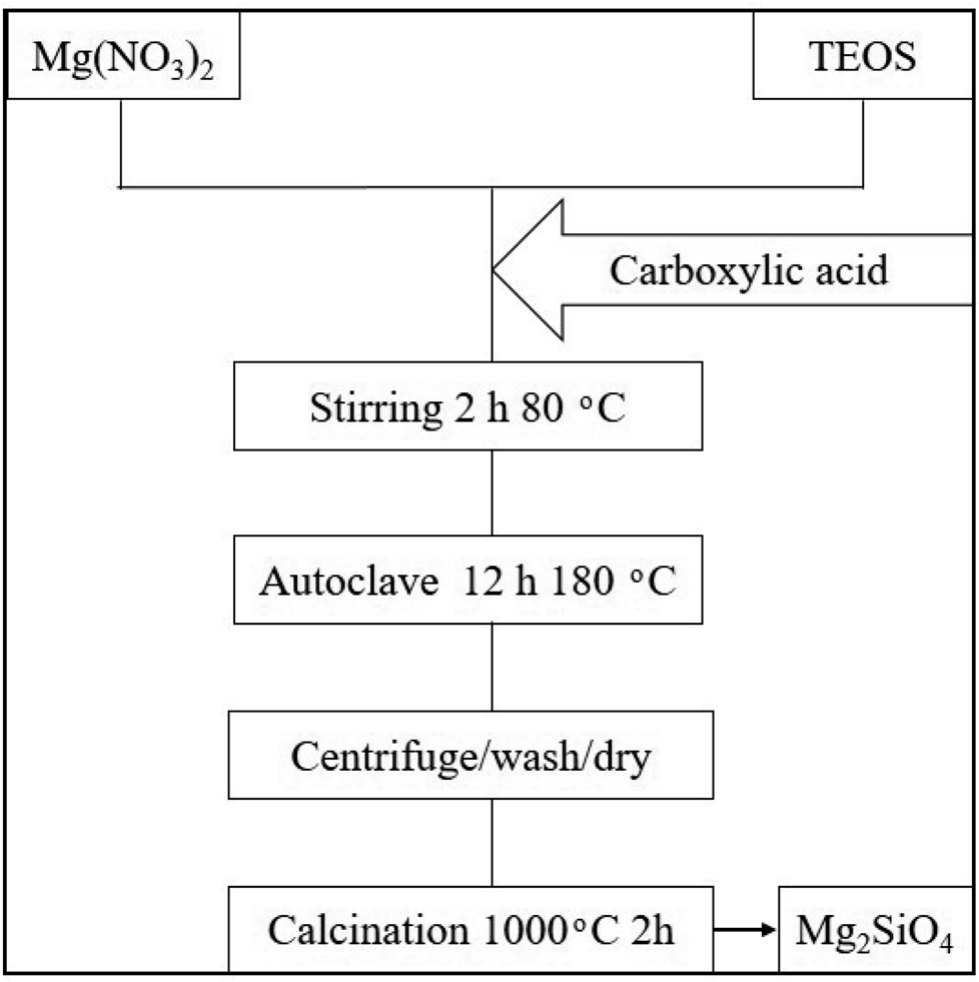
Flow diagram of Mg_2_SiO_4_ preparation.

In this study, Mg_2_SiO_4_ samples have been synthesized by the hydrothermal method, using Mg(NO_3_)_2_·6H_2_O, tetraethyl orthosilicate (TEOS), and organic acids. The overall flow diagram for the formation of forsterite is shown in Scheme 1. According to this figure, the Mg precursor (2 mmol) and organic acids (1.5–3 mmol) were blended with the proper amount of deionized water. Further, the ethanolic solution of TEOS (1 mmol) was dropped in the as-prepared solution and stirred vigorously for 2 hours at 80 °C. Afterward, the solution was placed in a 50 mL beaker, then transferred to a stainless steel autoclave and heated for 12 hours at 180 °C. The resulting precipitate was collected by a centrifuge process and washed five times with water and ethanol, then dried at 80 °C in the oven. Finally, the as-prepared white powder was further calcined at 1000 °C for 2 h in the muffle furnace. The different experimental conditions are shown in Table 1.

**Table 1:** Details of the Mg_2_SiO_4_ synthesis in the experimental section

Sample no.	Carboxylic acid (CA) agent	Ratio of CA : metal	Calcination temp.
S1	Succinic acid	1.5 : 1	1000 °C
S2	Succinic acid	3 : 1	1000 °C
S3	Citric acid	1.5 : 1	1000 °C
S4	Citric acid	3 : 1	1000 °C
S5	Maleic acid	1.5 : 1	1000 °C
S6	Maleic acid	3 : 1	1000 °C

### 2.3 Photodegradation of azo dyes

The photocatalytic degradation was performed in a homespun glass reactor system containing 200 mL of the dye solution with high concentration (20 ppm) at pH = 3, and 0.005 g L^−1^ of Mg_2_SiO_4_ nanopowder under ultraviolet (UV) and visible (Vis) light. The suspension was kept in dark conditions for 30 min with magnetic stirring (500 rpm) at room temperature (25 °C). Afterward, the system was illuminated by the UV or Vis lamp. The distances between the solution and the UV and Vis lamps were 40 and 25 cm, respectively. The as-prepared samples were washed, filtered, and centrifuged to detach the catalyst. Thereafter, the samples were analyzed with the UV-Vis spectrometer. The percentage of water pollutant photocatalytic degradation was measured as follows,^29,30^ where *A*_0_ and *A*_*t*_ are the concentrations of the water polluter at 0 and *t* min, respectively, by a UV-Vis spectrometer (eqn (1)).

1 Degradation % = [(*A*_0_ − *A*_*t*_)/*A*_0_] × 100

**Fig. 1:**
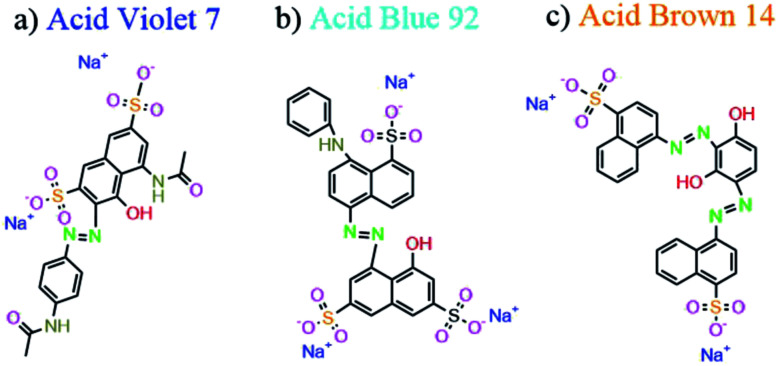
Chemical structures of (a) Acid Violet 7, (b) Acid Blue 92 and (c) Acid Brown 14.

Fig. 1 shows the chemical structures of Acid Violet 7, Acid Blue 92, and Acid Brown 14 which are the industrial dyes. Azo groups (N=N) are the main functional groups of these dyes.

## 3. Results and discussion

**Scheme 2:**
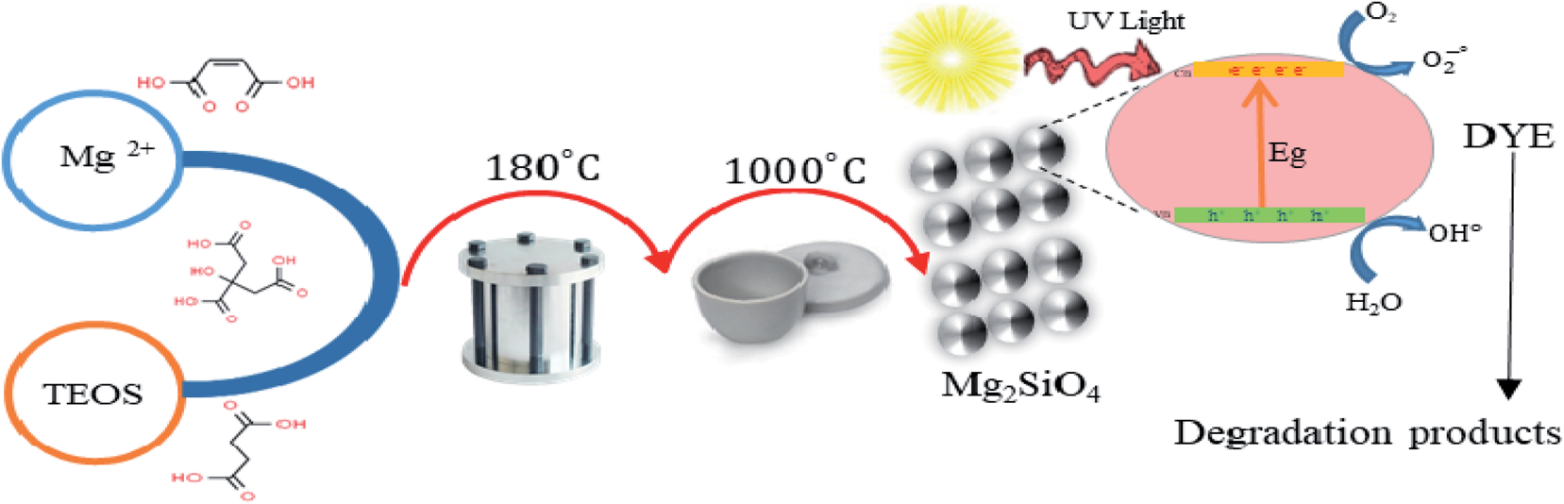
Schematic diagram of preparation and photocatalytic activity of Mg_2_SiO_4_.

This paper demonstrates the preparation of the nanostructured Mg_2_SiO_4_ as an ultraviolet light-sensitive photocatalyst with the assistance of carboxylic acids as a capping agent for the first time (Scheme 2). We studied the effect of synthesis parameters, such as the ratio of carboxylic acids to central metal ion, on the purity, shape, and chemical and physical properties of the Mg_2_SiO_4_ nanostructures. The main reason for using a carboxylic acid is the carboxylate group, which has an active site conjugated with Mg^2+^ ions and restricts the aggregation of Mg_2_SiO_4_ nanoparticles. Carboxylic acids are an efficient stabilizer for Mg_2_SiO_4_ nanostructures. Carboxyl and carbonyl groups are the main functional groups in the carboxylic acids. The chelating role of these groups with metal ions causes steric effects around the fabricated particles and prevents the aggregation.^31,32^

**Fig. 2:**
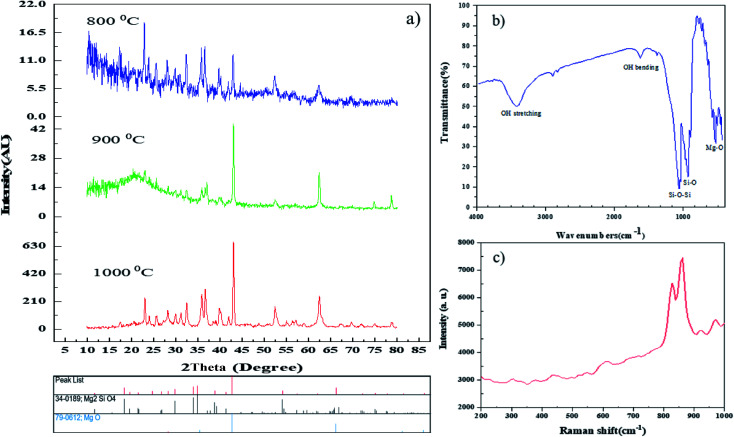
(a) XRD patterns of sample S1 at different temperatures, (b) FT-IR spectrum, and (c) Raman spectrum of the Mg_2_SiO_4_ nanostructures synthesized using succinic acid at 1000 °C.

The synthesized samples were characterized using different techniques. One of the best primary techniques in nanoparticle identification is X-ray diffraction (XRD) analysis which is appropriate and suitable for determining the crystalline structure and was utilized to examine the purity of the as-prepared samples. Fig. 2a displays the XRD patterns of the Mg_2_SiO_4_ nanostructure (sample S1) fabricated by the hydrothermal method using succinic acid as the capping agent at different temperatures. The XRD pattern of the as-prepared Mg_2_SiO_4_ powder is listed as a pure orthorhombic phase (space group: *Pmnb*, space group number: 62), which is close to the literature values (JCPDS no. 34-0189) and the sharp and intense peaks indicate the good crystallinity of the sample, with an impurity that corresponds to MgO with 79-0612 standard card number. By increasing the temperature of calcination, the intensity and purity of MgO at 2*θ* = 42° increased. The average crystallite size values are also calculated using the Scherrer equation (eqn (2)).

2 *D*_c_ = *Kλ*/*β* cos *θ*

where *β* is FWHM (full width at half maximum), *K* is the so-called shape factor, which usually takes a value of about 0.9, *λ* is the wavelength of the X-ray source used in XRD and *θ* is the diffraction angle.^33^ The computed crystallite size of these products was calculated by Scherrer’s equation in X’pert software by averaging the highest peaks in the XRD patterns. The average sizes of crystallites in the 800 °C, 900 °C, and 1000 °C patterns were calculated as 31 nm, 36 nm, and 29 nm, respectively. Fig. 2b shows the FT-IR spectrum of Mg_2_SiO_4_ (S1). The IR bands traced are represented in Table 2 with the corresponding functional groups. The FT-IR spectrum was used to follow the formation of Si–O–Si, MgO_6_, and MgO bands. The broad band peaks at 3435 cm^−1^ and 1633 cm^−1^ are correlated to the stretching and bending vibrations of the surface OH group, respectively. The bands at 1095 cm^−1^ and 953 cm^−1^ are the Si–O–Si and Si–O stretching, and the peak at 895 cm^−1^ is related to the SiO_4_ bending vibration. According to this spectrum, the two bands at 427 cm^−1^ and 777 cm^−1^ are assigned to the vibrations of octahedral MgO_6_ and the band at around 551 cm^−1^ relates to the Mg–O bond.^34^

**Table 2:** Traced functional groups in the FT-IR spectrum of sample S1

Traced functional group	Wave no.
MgO_6_ octahedral	427 cm^−1^
Mg–O bending	551 cm^−1^
MgO_6_ octahedral	777 cm^−1^
Si–O bending	895 cm^−1^
Si–O stretching	953 cm^−1^
Si–O–Si	1095 cm^−1^
Surface OH bending	1633 cm^−1^
Surface OH stretching	3435 cm^−1^

The Raman spectrum of Mg_2_SiO_4_ nanoparticles is shown in Fig. 2c. The spectrum was measured in the spectral region from 200–1000 cm^−1^. The narrow width and the intensity of the peaks demonstrate the excellent quality and homogeneity of the as-prepared Mg_2_SiO_4_ nanoparticles which confirm the XRD analysis and EDS spectroscopy results. As shown in Fig. 2c, the frequencies lower than 450 cm^−1^ were attributed to the Mg–O stretching force and O–O repulsive force among rigid translation of the SiO_4_ tetrahedral. Moreover, the frequencies greater than 450 cm^−1^ such as 530, 617, 819 and 857 cm^−1^ are assigned to Si–O stretching.^35^ It has been observed that the Raman and infrared modes show considerable agreement with each other.

**Fig. 3:**
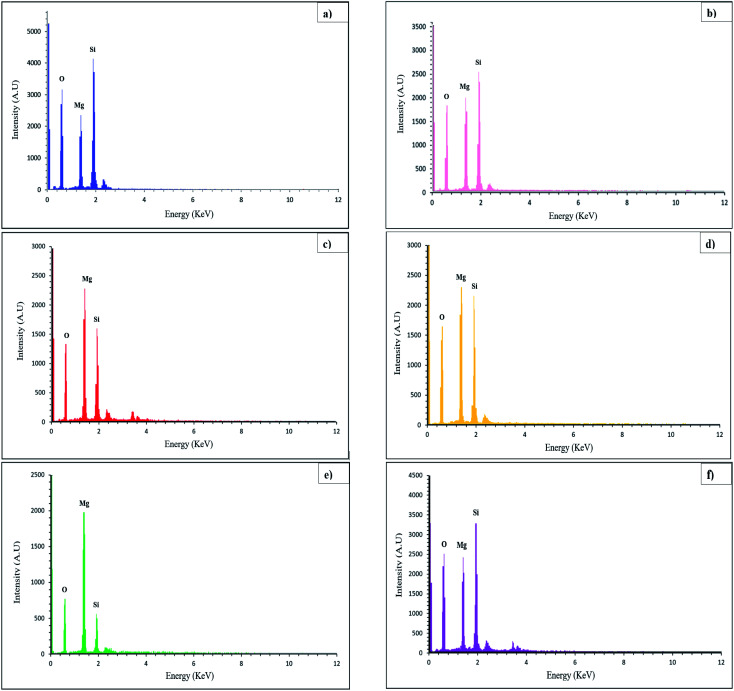
EDS results of (a) sample S1, (b) sample S2, (c) sample S3, (d) sample S4, (e) sample S5, and (f) sample S6. The results confirm the presence of Mg, Si, and O atoms in Mg_2_SiO_4_.

EDS analysis can be used to identify the elemental composition of the surface of materials by detecting elements. EDS spectra of Mg_2_SiO_4_ nanoparticles (sample S1–S6) are exhibited in Fig. 3. The peaks of Mg, Si, and O elements are in the defined energy of them, and other peaks were not detected, so the purity of the product was confirmed. The atomic percentages of the three elements magnesium, silicon, and iron are listed in Table 3. The calculated surface elemental composition was altered from the bulk composition. Nevertheless, from the XRD data the formation of magnesium silicate was confirmed.

**Table 3:** Atomic percentages of the surface composition of the Mg_2_SiO_4_ samples

Sample no.	*A*% of Mg	*A*% of Si	*A*% of O
S1	11.30	15.45	73.25
S2	15.28	16.24	68.47
S3	20.53	13.60	65.87
S4	23.02	21.18	55.81
S5	36.73	11.55	51.71
S6	13.46	14.85	71.69

In order to study the effect of the capping agent on the morphology and size distribution of Mg_2_SiO_4_ nanostructures, scanning electron microscopy (SEM) was carried out. The morphology and the size of the Mg_2_SiO_4_ nanoparticles were noticeably affected by the different preparation conditions.

**Fig. 4:**
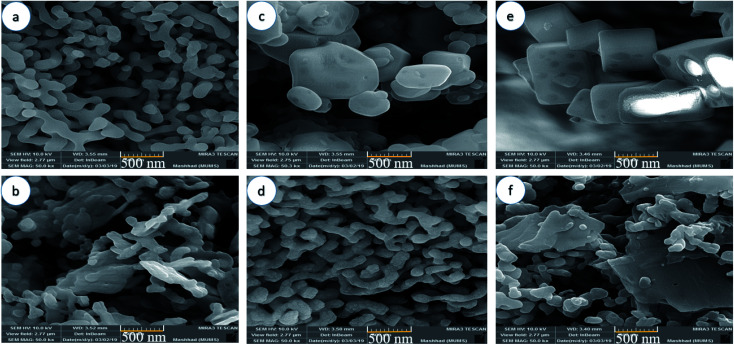
SEM images of samples (a) S1, (b) S2, (c) S3, (d) S4, (e) S5, and (f) S6 synthesized using different carboxylic acids as the capping agent.

Fig. 4a and b show the SEM images of the Mg_2_SiO_4_ particles utilizing succinic acid as the shape modifier. The steric effect and surface adsorption of succinic acid on the particles favored the formation of a narrow size distribution for the Mg_2_SiO_4_. By raising the amount of succinic acid, the shape of particles varied to an aggregated form. The high acidity of the succinic acid is the main environmental condition that influenced the morphology and induced aggregation (Fig. 4b). When changing the ratio of succinic acid to central metal ion from 1.5 to 3, the average size of Mg_2_SiO_4_ nanotubes altered from about 150 nm to 100 nm. The excess amount of succinic acid caused smaller nanotubes but induced aggregation.

Fig. 4c and d show the SEM images of the Mg_2_SiO_4_ nanostructures fabricated using citric acid as the morphology modifying agent. As presented in the figure, the Mg_2_SiO_4_ remarkably changed when increasing the ratio of citric acid to magnesium nitrate from 1.5 to 3. Citric acid has three carboxylic (–COOH) groups and one hydroxyl (–OH) group. Due to the coordinating influence of the carboxylic group with Mg^2+^ ions and their adsorption on the surface of the Mg_2_SiO_4_ structures, the morphology changed from an aggregated shape (Fig. 4c) to a worm-like form (Fig. 4d). The particles generally are well faceted and exhibit a profoundly symmetric shape, showing that they are monocrystalline. By adding twice the amount of citric acid to the medium, the size of the nano-shaped Mg_2_SiO_4_ reduced to 140 nm approximately.

Fig. 4e and f display the SEM images of the Mg_2_SiO_4_ nanostructures fabricated using maleic acid. Various morphologies were obtained using the different ratios of maleic acid to magnesium source. By increasing the concentration of maleic acid, the morphology of Mg_2_SiO_4_ shifted from a hollow cubic nanostructure (Fig. 4e) to a nanosheet and the size of particles changed from 0.48 μm to 89 nm. Therefore, a high concentration of maleic acid caused aggregation (Fig. 4f) and also decreased the catalytic activity. The high concentration of maleic acid in the reaction medium might provide an excess nucleation process which causes aggregation. Using SEM showed that sample S4 is the optimized sample, due the good size, shape and uniformity.

**Fig. 5:**
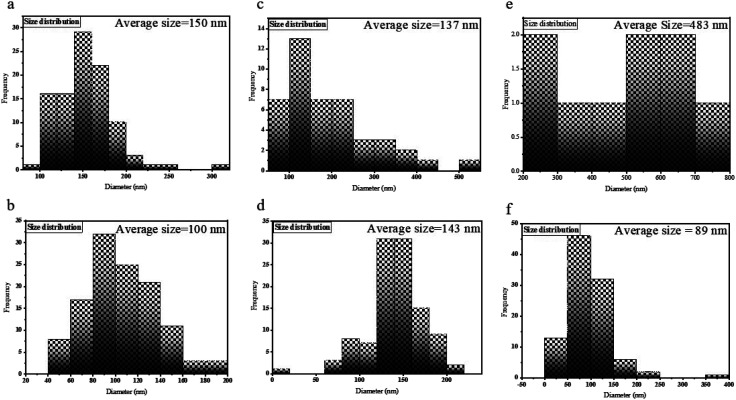
The size distributions of samples (a) S1, (b) S2, (c) S3, (d) S4, (e) S5, and (f) S6.

Fig. 5 manifests the size distributions of the samples calculated from their SEM images using Digimizer (image analyzer) software. As indicated in Fig. 5a, most particles are found in the range of 100–150 nm. In Fig. 5b, the average size was 100 nm. Fig. 5c shows the size distribution of sample S3, and most of the particles have a size of about 100 nm. The size distribution of sample S4 is shown in Fig. 5d. The range of sizes was 90–200 nm. The size distribution of sample S5 is between 200–700 nm (Fig. 5e). Fig. 5f shows that the average size of nanoparticles is between 60 and 100 nm.

**Fig. 6:**
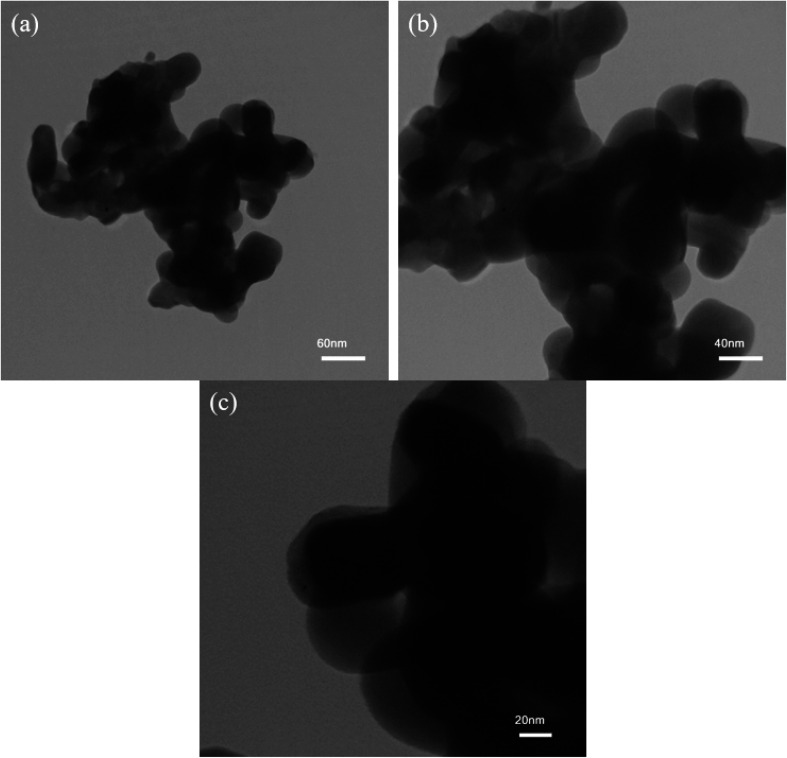
TEM images of Mg_2_SiO_4_ nanostructure (sample S1) as the appropriate sample.

Fig. 6 depicts TEM images of optimum Mg_2_SiO_4_ nanoparticles synthesized using Mg(NO_3_)_2_ and tetraethyl orthosilicate in the presence of succinic acid (sample S1). The TEM images corroborate that the as-prepared sample has been formed from plate-like nanoparticles. This is consistent with the SEM images of this Mg_2_SiO_4_ nanostructure.

Photocatalytic activity is reliant on the bandgap of the nanostructure and then the light absorption capacity of a photocatalyst. The optical bandgap can be determined using the Tauc equation (eqn (3)).

**Fig. 7:**
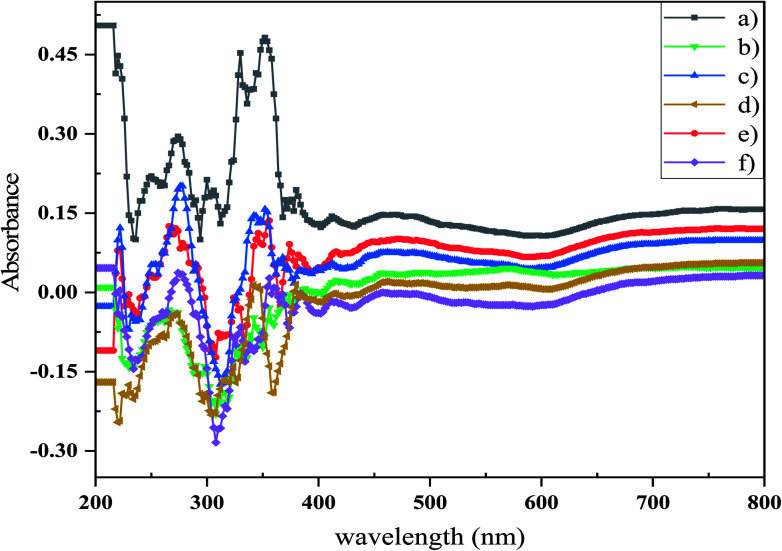
DRS spectra of (a) sample S1, (b) sample S2, (c) sample S3, (d) sample S4, (e) sample S5, and (f) sample S6. These results showed that the type and concentration of capping agent considerably changed the optical properties of the Mg_2_SiO_4_ nanostructures.

**Fig. 8:**
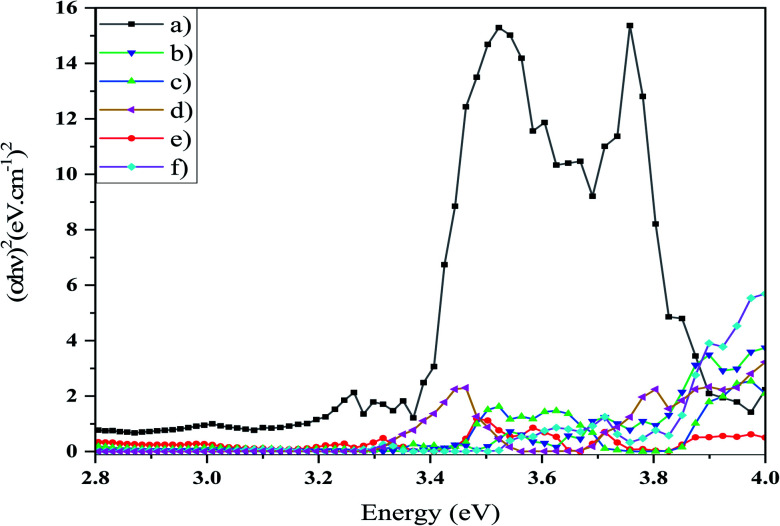
Curve (*αhν*)^2^ against *hν* of (a) sample S1, (b) sample S2, (c) sample S3, (d) sample S4, (e) sample S5, and (f) sample S6. The calculated bandgap value was measured through extrapolating.

3 (*αhν*)^*n*^ = [*B*(*hν* − *E*_g_)]

where *hν* is the energy of the photon, *α* is the absorption coefficient, *B* is the constant relative to the material, and *n* is either 2 for a direct transition or 1/2 for an indirect transition. UV-Vis absorption spectra (DRS) of the synthesized photocatalysts, *i.e*. S1, S2, S3, S4, S5, and S6, are shown in Fig. 7. The weak absorption band at lower wavelength may be assigned to the metastable states developed between the valence band and conduction band^36^ and shows two excitonic absorption peaks at 273 and 352 nm. The absorption peak that occurs at 352 nm is due to the fundamental absorption within the bandgap of this material. Other absorption bands around 300 and 330 nm were attributed to the impurities or vacancies.^37^ The bandgap can be measured based on the absorption spectrum using the Tauc equation. The bandgaps of samples were calculated through targeting and extrapolating to the energy axis the linear section of the plot (*αhν*)^2^ against *hν* (Fig. 8). The bandgap energy values of the Mg_2_SiO_4_ samples are listed in Table 4. From the *E*_g_ values of the as-prepared Mg_2_SiO_4_ samples, it is observed that magnesium silicate can be applied as a photocatalyst in dye degradation.

**Table 4:** Calculated bandgaps of samples S1, S2, S3, S4, S5, and S6

Sample no.	Bandgap energy (eV)
S1	3.38
S2	3.78
S3	3.44
S4	3.78
S5	3.44
S6	3.81

**Fig. 9:**
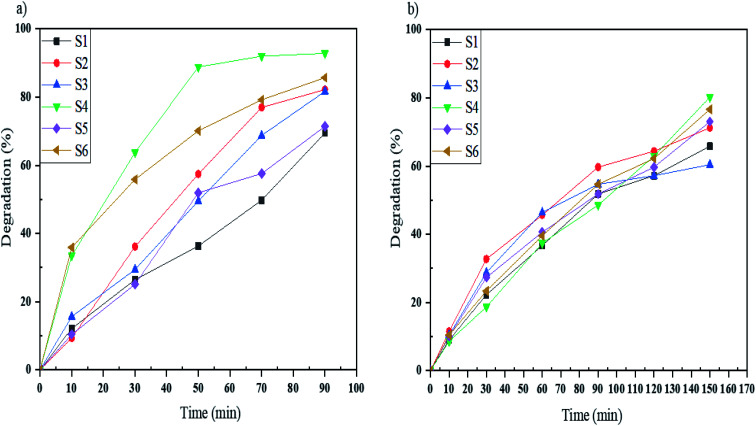
Photocatalytic degradation of Acid Violet 7 under (a) UV, and (b) Vis light irradiation using samples S1, S2, S3, S4, S5, and S6.

In this work, the photocatalytic activity of Mg_2_SiO_4_ was investigated by the degradation of three particular azo dyes (as water contaminants from industries) under UV and Vis light. As demonstrated in Fig. 9, the photodegradation of Acid Violet 7 under ultraviolet and visible light irradiation for the defined time was done. By using the photocatalytic calculations (eqn (1)), the percentages of photodegradation under UV irradiation were 62, 82, 81, 92, 71, and 85% for sample S1, sample S2, sample S3, sample S4, sample S5, and sample S6, respectively (Fig. 9a) and the photodegradation percentages for Acid Violet 7 under Vis light were 65% by S1, 71% by S2, 60% by S3, 80% by S4, 73% by S5, and 76% by S6 (Fig. 9b). Sample S4 had the highest efficiency under UV and Vis light. This high percentage of photodegradation could happen because of the uniform shape and smaller size of the Mg_2_SiO_4_ particles compared to the other samples.

**Fig. 10:**
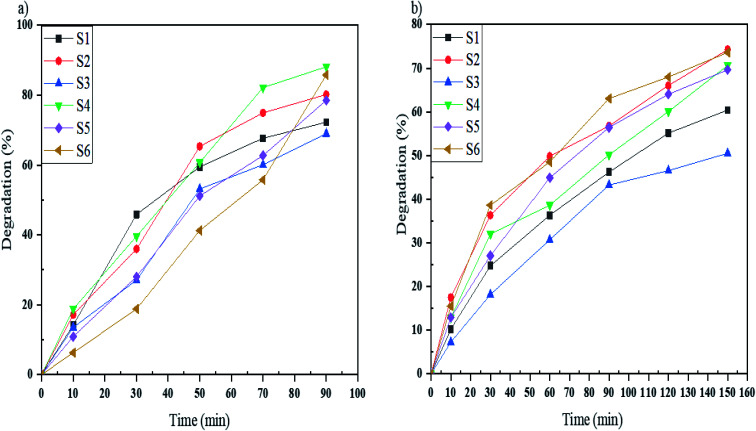
Photocatalytic degradation of Acid Blue 92 under (a) UV, and (b) Vis light irradiation using samples S1, S2, S3, S4, S5, and S6.

Acid Blue 92 is a monoazo dye that was degraded by samples S1–S6 under UV and Vis light in the defined time. As shown in Fig. 10a, samples S1–S6 degrade 72, 80, 69, 88, 78 and 85%, respectively, in 90 minutes under UV irradiation, and in Fig. 10b, the degradation percentages of Acid Blue 92 under Vis light were 60% for S1, 74% for S2, 50% for S3, 70% for S4, 69% for S5, and 73% for S6. Sample S4 under UV and sample S2 under Vis irradiation had the highest percentages. The comparable bandgaps of these samples and appropriate morphologies increase the photodegradation of Acid Blue 92.

**Fig. 11:**
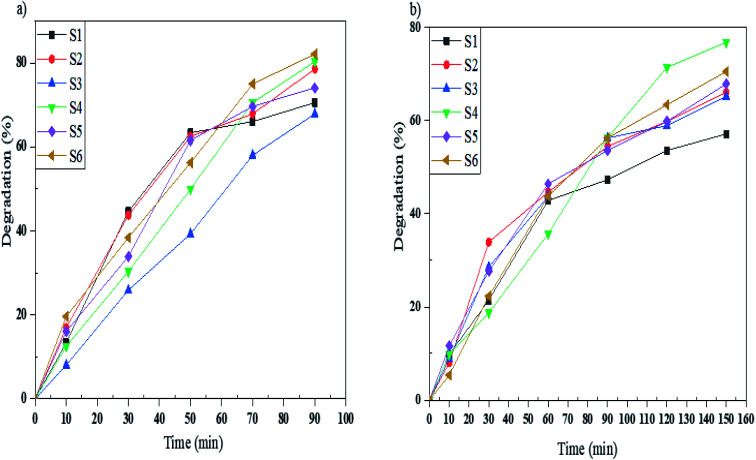
Photocatalytic degradation of Acid Brown 14 under (a) UV, and (b) Vis light irradiation using samples S1, S2, S3, S4, S5, and S6.

To study the photocatalytic activity of Mg_2_SiO_4_ nanostructures in the degradation of water pollutants, we studied the photodegradation of Acid Brown 14 as a diazo dye. Sample S6 gave the highest percentage of degradation under UV light and, also sample S4 under Vis light gave the maximum percentage of degradation which are 82 and 76%, respectively (Fig. 11a and b). Furthermore the excessive coverage of photocatalyst with dye molecules can remarkably decrease the photodegradation percentage under UV and Vis irradiation.

The other proof is the consumption of reactive radicals due to interactions with degradation products, so fewer radicals are ready for dye photodegradation. This result is intensified due to the point that dye molecules should be adsorbed at the surface of the catalyst to be degraded by light irradiation, so degradation byproducts are produced very near to the active catalyst area and have a large probability to take part in reactions with radicals.

The assumed mechanism of photodegradation of azo dyes by Mg_2_SiO_4_ nanoparticles can be shown as:^38^Mg_2_SiO_4_ nanostructures + *hν* → Mg_2_SiO_4_ nanostructures* + e^−^ + h^+^h^+^ + H_2_O → OH˙ + H^+^2h^+^ + 2H_2_O → H_2_O_2_ + 2H^+^H_2_O_2_ → 2OH˙e^−^ + O_2_ → O_2_^−^˙O_2_^−^˙ + 2OH˙ + H^+^ → H_2_O_2_ + O_2_H_2_O_2_ → 2OH˙O_2_^−^˙ + azo dye → degraded productsIt seems that the best photocatalytic performance of Mg_2_SiO_4_ is with sample S4, which is synthesized in the presence of citric acid. It is usually assumed that the recombination of electrons (e^−^) and holes (h^+^) has a notable impression on the photocatalytic activity and decreasing the recombination can increase the photocatalytic performance.^39^ The proper bandgap and uniform size of sample S4 decrease the chance of electron–hole recombination compared to the other samples relatively. Also, as shown in the XRD patterns in Fig. 2, the excessive amount of MgO in the chemical structure might assist the Mg_2_SiO_4_ nanostructures by providing extra surface area, therefore more photocatalytic activity.

**Fig. 12:**
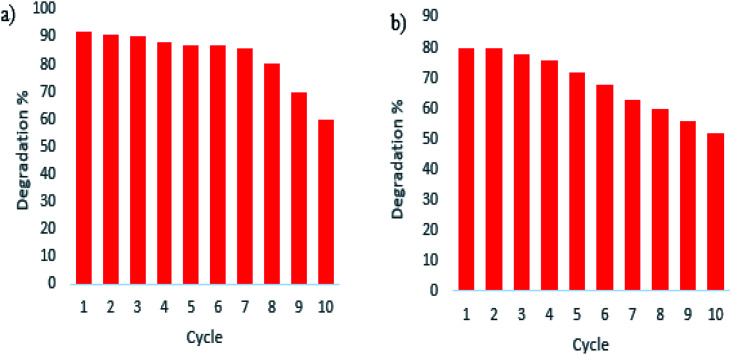
Recycling and reusing of optimized sample S4 in the degradation of Acid Violet 7 under (a) UV and (b) Vis light irradiation.

To prove the stability of the photocatalyst under dye degradation, the photocatalytic reaction of the optimized sample over ten successive cycles of photocatalytic reaction has been examined. According to the experimental results, as shown in Fig. 12, the photocatalytic performance of the optimized sample under UV and visible irradiations decreased only slightly after seven cycles of the photocatalytic reaction in optimum conditions which shows the excellent stability of the as-prepared sample.

**Scheme 3:**
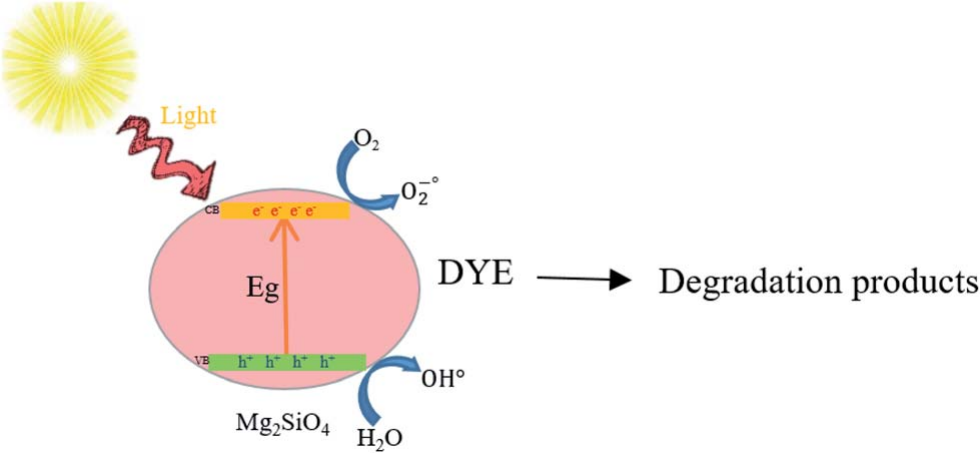
Schematic diagram of the reaction mechanism of azo dye photodegradation by Mg_2_SiO_4_.

Scheme 3 shows the photodegradation process of azo dyes using Mg_2_SiO_4_ nanostructures. It is conspicuous, in the proximity of light, that the photocatalyst can generate an electron–hole pair in the conduction and valance band that effected degradation of contaminants.^40–48^

## 4. Conclusion

In conclusion, Mg_2_SiO_4_ nanostructures with various morphologies have been successfully fabricated *via* a hydrothermal–calcination approach. As novel capping agents, carboxylic acids (CA) were used in the presence of TEOS and Mg(NO_3_)_2_ to synthesise Mg_2_SiO_4_. To obtain the good and uniform shape of forsterite, various parameters were studied. Different ratios of CA agent to central ion have been examined and the sample S4 was chosen as the optimum sample. Mg_2_SiO_4_ nanostructures were used as photocatalysts to treat water containing azo dyes. Maximum photodegradation values for Acid Violet 7 under UV and Vis light were 92 and 80%, respectively. Also, for Acid Blue 92 under UV and Vis irradiation the values were 88 and 74%. Finally the percentages of photodegradation for Acid Brown 14 as a diazo dye under UV and Vis light were about 82 and 76%.

## Conflicts of interest

The authors declare that there are no conflicts of interest regarding the publication of this manuscript.

## Supplementary Material
